# The complete mitochondrial genome of the five-lined cardinalfish *Cheilodipterus quinquelineatus* (Apogonidae)

**DOI:** 10.1080/23802359.2018.1467221

**Published:** 2018-04-26

**Authors:** Ambrocio Melvin Matias, James Hereward

**Affiliations:** a School of Biological Sciences, The University of Queensland, St Lucia, Australia;; b Institute of Biology, University of the Philippines Diliman, Quezon City, Philippines

**Keywords:** Cardinalfish, mitogenome, Philippines

## Abstract

*Cheilopdipterus quinquelineatus* (Apogonidae) is an interesting species for investigating processes driving population divergence in marine system due to its wide distribution and life-history traits. However, to date, there is a limited genetic resource available for this species, or the family Apogonidae as exemplified by the availability of only two mitogenomes. In this study, we assembled the whole mitochondrial genome of this species yielding a 16,537 bp circular assembly composed of the typical vertebrate mitochondrial features. Phylogenetic inference of the supraordinal group of *C. quiquelineatus* showed monophyly of the major families.

The five-lined cardinalfish *Cheilopdipterus quinquelineatus* (Apogonidae) is a widely distributed reef fish present across the Indo-West Pacific. This species is noted for its mouth-brooding reproductive behaviour, wherein males incubate the eggs in their mouth after fertilization. This characteristic likely contributes to the high population genetic structuring typically observed in species belonging to Apogonidae (Hoffman et al. 2005), which in turn could result in cryptic species within the family. For example, we observed high divergence in the *COI* of *C. quinquelineatus* obtained from the Philippines (Matias, unpublished data). Although all of these *COI* haplotypes matched previously published DNA barcodes for *C. quinquelineatus* (Hubert et al. 2012), they still separate into two divergent clades, thereby indicating the possibility of cryptic species or a possible misidentification. These features make *C. quinquelineatus* ideal for studying evolutionary processes that contribute to divergence in marine system; but genetic resources for this species, or Apogonidae in general, is presently lacking, as exemplified by the presence of only two mitogenomes sequenced for the family.

Here, we assembled the mitogenome of *C. quinquelineatus* sampled from Bolinao, Philippines (16.36° latitude, 119.85° longitude) and is deposited in the Institute of Biology, University of the Philippines Diliman with identifier BPP5113. Using the genomic DNA extracted from fin clips (DNA extract BPP5113 stored in the School of Biological Sciences, University of Queensland) with the OMEGA E-Z 96 Tissue DNA Kit (Omega Bio-tek, Norcross, GA), a genomic library with insert size of ∼380 bp was prepared using NeBNext Ultra DNA Kit (New England Biolabs, Ipswich, MA) and paired-end sequenced (150 bp) by AnnoRoad (Beijing, China). For the mitogenome assembly, we first mapped the reads to the *Apogon semilineatus* mitogenome (Miya et al. 2013) in Geneious v10.2.3 (Kearse et al. 2012). The mapped reads were then recursively aligned to form contiguous consensus until the circular mitogenome was resolved. The resulting consensus was annotated using MitoAnnotator (Iwasaki et al. 2013). We then examined the evolution of mitochondria in the supraordinal group Gobiomorpharia (Betancur-R et al. 2013) by inferring the phylogenetic relationships of mitogenomes from selected Gobiomorpharia genera.

The complete mitogenome of *C. quinquelineatus* is 16,537 bp in length with a GC content of 47% (GenBank accession number MH102356). It is composed of the typical vertebrate mitochondrial features, which include two ribosomal RNAs (rRNAs) genes, 22 transfer RNAs (tRNAs) genes, a control region, and 13 protein coding genes (pcg) that all start with ATG except for the *COI* that starts with GTG. From the inferred phylogeny based on mitogenomes of selected Gobiomorpharia genera (Figure 1), the mitogenome of *C. quinquelineatus* clusters with the other two Apogonidae mitogenomes resulting to a monophyletic Apogonidae, which is sister to the family Kurtidae mitogenome (Betancur-R et al. 2013). Similarly, each of the other major families of Gobiomorpharia formed a monophyletic clade, yielding a topology consistent with the suggested Gobiiformes order by Thacker (2014) (Figure 1).

**Figure 1.**
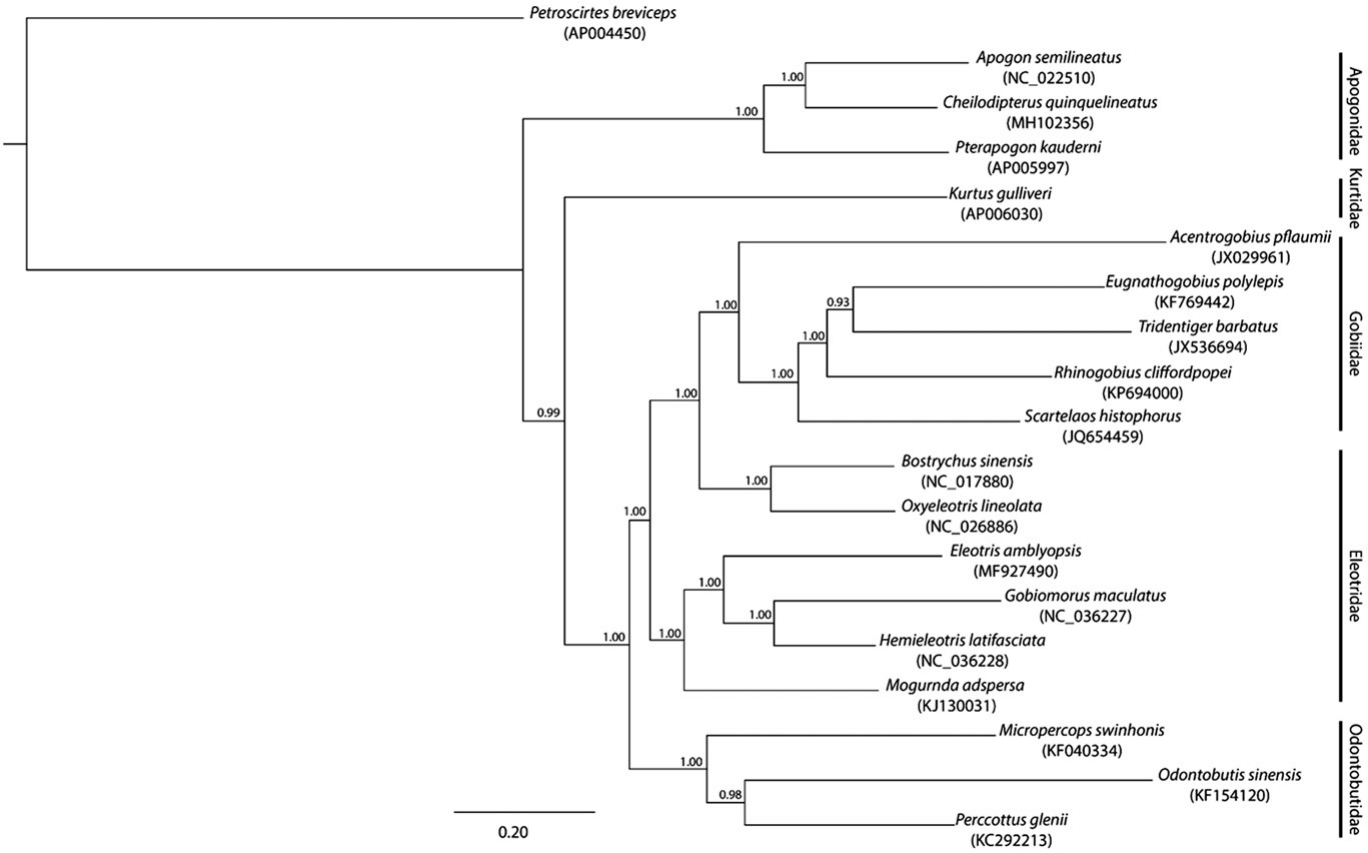
The phylogenetic relationship of selected Gobiomorpharia genera inferred from the whole mitogenome using a Bayesian inference with GTR model of DNA substitution as implemented in MrBayes v3.2.6 (Hulsenbeck and Ronquist 2001). The tips of the tree show the species name and GenBank accession number of the mitogenomes, while the families are indicated on the right-hand side of the figure. The posterior probability of each branch is denoted by the number in the nodes. The species *Petroscirtes breviceps* was used as an outgroup.
